# Affective Voice Interaction and Artificial Intelligence: A Research Study on the Acoustic Features of Gender and the Emotional States of the PAD Model

**DOI:** 10.3389/fpsyg.2021.664925

**Published:** 2021-05-04

**Authors:** Kuo-Liang Huang, Sheng-Feng Duan, Xi Lyu

**Affiliations:** ^1^Department of Industrial Design, Design Academy, Sichuan Fine Arts Institute, Chongqing, China; ^2^Department of Digital Media Art, Design Academy, Sichuan Fine Arts Institute, Chongqing, China

**Keywords:** voice-user interface (VUI), affective computing, acoustic features, emotion analysis, PAD model

## Abstract

New types of artificial intelligence products are gradually transferring to voice interaction modes with the demand for intelligent products expanding from communication to recognizing users' emotions and instantaneous feedback. At present, affective acoustic models are constructed through deep learning and abstracted into a mathematical model, making computers learn from data and equipping them with prediction abilities. Although this method can result in accurate predictions, it has a limitation in that it lacks explanatory capability; there is an urgent need for an empirical study of the connection between acoustic features and psychology as the theoretical basis for the adjustment of model parameters. Accordingly, this study focuses on exploring the differences between seven major “acoustic features” and their physical characteristics during voice interaction with the recognition and expression of “gender” and “emotional states of the pleasure-arousal-dominance (PAD) model.” In this study, 31 females and 31 males aged between 21 and 60 were invited using the stratified random sampling method for the audio recording of different emotions. Subsequently, parameter values of acoustic features were extracted using Praat voice software. Finally, parameter values were analyzed using a Two-way ANOVA, mixed-design analysis in SPSS software. Results show that gender and emotional states of the PAD model vary among seven major acoustic features. Moreover, their difference values and rankings also vary. The research conclusions lay a theoretical foundation for AI emotional voice interaction and solve deep learning's current dilemma in emotional recognition and parameter optimization of the emotional synthesis model due to the lack of explanatory power.

## Introduction

Nowadays, the core technologies of artificial intelligence (AI) are becoming increasingly mature. People face a new bottleneck in giving the “emotional temperature of humans” to a cold, intelligent device (Yonck, 2017). The conversational voice-user interface (VUI) is the most natural and instinctive interactive mode for humans. Recently, natural language processing (NLP) has improved significantly due to the development of deep learning (DL) technology. The VUI demands of the new type of intelligent products transform communication to include emotional listening and feedback of users (Hirschberg and Manning, 2015; Dale, 2016; Chkroun and Azaria, 2019; Harper, 2019; Nguyen et al., 2019; Guo et al., 2020; Hildebrand et al., 2020). Giving computers similar emotional mechanisms and emotional intelligence concepts as humans is becoming increasingly critical in the information and cognitive sciences. The goal of “affective computing” is to endow computers with abilities of understanding and generating affective characteristics. Finally, the computer can become intimate with the nature and makeup of vivid interactions, like people. This involves interdisciplinary study in the areas of psychology, sociology, information science, and physiology (Picard, 2003, 2010) and is becoming a hot spot of laboratory research in academic and industrial circles (Bänziger et al., 2015; Özseven, 2018). Although VUI has considerable potential, effective semantic and emotional communication not only requires the subtle understanding of the physics and psychology of voice signals but also needs a method of extracting and analyzing voice features from human voice data (Picard, 2003; Guo et al., 2020; Hildebrand et al., 2020).

Affective computing is crucial to implementing man–machine emotional interactions through intelligent products (Picard, 2010; Dale, 2016). In the past, many studies of emotional voice recognition and synthesis have been reported. Nevertheless, they mainly establish acoustic models and systems based on information science. Abundant voice data have been input into the DL core of AI and several affective factors of acoustic features summarized from the 3-D pleasure-arousal-dominance (PAD) emotional state model on a “continuous dimension.” A mathematical model was constructed and abstracted using mathematical knowledge and computer algorithms. Subsequently, the computer was able to learn from the data and make predictions by combining training data and its large-scale operation capability (Ribeiro et al., 2016; Rukavina et al., 2016; Kratzwald et al., 2018; Vempala and Russo, 2018; Badshah et al., 2019; Heracleous and Yoneyama, 2019; Guo et al., 2020). Although these practices can gain accurate prediction results quickly, they do not provide an understanding of where the results come from (e.g., black box) and lack explanatory ability (Kim et al., 2016; Ribeiro et al., 2016; Murdoch et al., 2019; Molnar, 2020). As a result, understanding how to adjust the model parameters is a problem that has yet to be solved, requiring an urgent empirical study of the connection between acoustic features and psychology as the theoretical basis for adjustment of model parameters (Ribeiro et al., 2016; Skerry-Ryan et al., 2018; Evans et al., 2019; Molnar, 2020). Research into voice rhythms from the cognitive psychology perspective has mainly focused on fundamental frequency, sound intensity, voice length, and other features (Juslin and Scherer, 2005). Emotional classifications are described quantitatively, which is different from the “continuous dimension” in existing intelligent systems. None of these studies yields 3-D coordinates through transformation to provide affection matching.

As a result of these shortcomings, an empirical study on the correlation between information enabling the emotional evaluation of acoustic features concerning emotional voice state and psychology is required in AI emotional voice interaction using a PAD model, which is the theoretical basis for adjustment of model parameters (Ribeiro et al., 2016; Skerry-Ryan et al., 2018; Evans et al., 2019; Molnar, 2020). Different average speech characteristics between males and females in human conversations have been reported in most studies (Childers and Wu, 1991; Feldstein et al., 1993). Furthermore, males and females show different emotional expressions. This study connected emotional states and voice features of male and female users through cross informatics and cognitive psychology from the voice interaction application scenes of intelligent products. Hence, this study focuses on the influences of “gender” and “emotions” on the “physical features of voices” in human–computer interactions as well as the quantitative expressions of the “physical features of voices.” The research conclusions lay a theoretical foundation for AI emotional voice interaction and solve DL's current dilemma in emotional recognition and parameter optimization of the emotional synthesis model due to lack of explanatory powers.

## Literature Review

### Studies on Emotions and Classification

According to research within psychology and the neurosciences, there is extensive interaction between the emotions and cognition of humans (Osuna et al., 2020), displaying behavioral and psychological features (Fiebig et al., 2020) that have a profound impact on the expression, tone, and posture behavior of people in daily life (Scherer, 2003; Ivanović et al., 2015; Poria et al., 2017). In the past 20 decades, studies on emotions have increased significantly (Wang et al., 2020). At present, there are two mainstream affective description modes. One is to make a qualitative description of an emotional classification using adjectives from the perspective of “discrete dimensions,” such as the six basic emotion categories proposed by Ekman and Oster (1979). The other is to describe the consequence determined by common affective factors of a “continuous dimension.” The emotional states can be characterized and divided by quantitative emotional coordinates on different dimensions (Sloman, 1999; Bitouk et al., 2010; Chauhan et al., 2011; Harmon-Jones et al., 2016; Badshah et al., 2019). Specifically, 1-D space focuses on positive or negative emotional classification, and 2-D spatial emotional states are generally expressed by two coordinates, such as peace–excitement and happiness–sadness. The 3-D space is proposed by Schlosberg (1954), Osgood (1966), Izard (1991), Wundt and Wozniak (1998), and Dai et al. (2015), respectively.

Quantitative measurement of emotions is a requirement of affective computing (Dai et al., 2015). Because three-dimensional space is easy to compute, computational models of emotion (CMEs) in the current AI system adopt the continuous dimension; the most used is the PAD model proposed by Mehrabian and Russell in 1994. The PAD model hypothesizes that users have three emotional states according to the situation stimulus, including pleasure, arousal, and dominance. These 3-D axes act as an emotional generation mechanism (Mehrabian and Russell, 1974; Wang et al., 2020). For example, emotions are divided into eight states with eight blocks of 3-D negative (–) and positive (+) combinations in the three dimensions as seen in Table 1 (Mehrabian, 1996b).

**Table 1:** Mapping of the eight Mehrabian basic emotions in PAD space.

	**Trait combination**	**Emotional state**
P (pleasure-displeasure): emotional state's positivity or negativity	+P+A+D	Exuberant
	–P–A–D	Bored
A (arousal-nonarousal): physical activity and mental alertness	+P+A–D	Dependent
	–P–A+D	Disdainful
D (dominance-submissiveness): feeling of control	+P–A+D	Relaxed
	–P+A–D	Anxious
	+P–A-D	Docile
	–P+A+D	Hostile

*Datasource: Mehrabian (1996b)*.

As a CME, PAD can distinguish different emotional states effectively (Russell, 1980; Gao et al., 2016) and break from the traditional tag-description method. As one of the relatively mature emotional models (Mehrabian and Russell, 1974; Mehrabian, 1996a; Gunes et al., 2011; Jia et al., 2011; Chen and Long, 2013; Gao et al., 2016; Osuna et al., 2020; Wang et al., 2020), the PAD model measures the mapping relationship between emotional states and typical emotions by “distance” to some extent, thus transforming the analytical studies of discrete emotional voices into quantitative studies of emotional voices (Mehrabian and Russell, 1974; Mehrabian, 1996a; Gunes et al., 2011; Jia et al., 2011; Chen and Long, 2013; Gao et al., 2016; Osuna et al., 2020; Wang et al., 2020). It has been extensively applied in information processing, emotional computing, and man–machine interaction (Dai et al., 2015; Weiguo and Hongman, 2019). PAD is beneficial for establishing an external stimulus emotional calculation model to realize emotional responses during personalized man–machine interaction (Weiguo and Hongman, 2019).

### Affective Computing and Emotions in Voice Interaction

Voice signals are the most natural method of communication for people (Weninger et al., 2013). On the one hand, voice signals contain the verbal content to be transmitted. On the other hand, rhythms in the vocalizations contain rich emotional indicators (Murray and Arnott, 1993; Gao et al., 2016; Noroozi et al., 2018; Skerry-Ryan et al., 2018). Each emotional state has unique acoustic features (Scherer et al., 1991; Weninger et al., 2013; Liu et al., 2018). For example, various prosodic features, including different tones, velocity, and volume, can express the speaker's different emotional states (Apple et al., 1979; Trouvain and Barry, 2000; Chen et al., 2012; Yanushevskaya et al., 2013).

**Figure 1:**
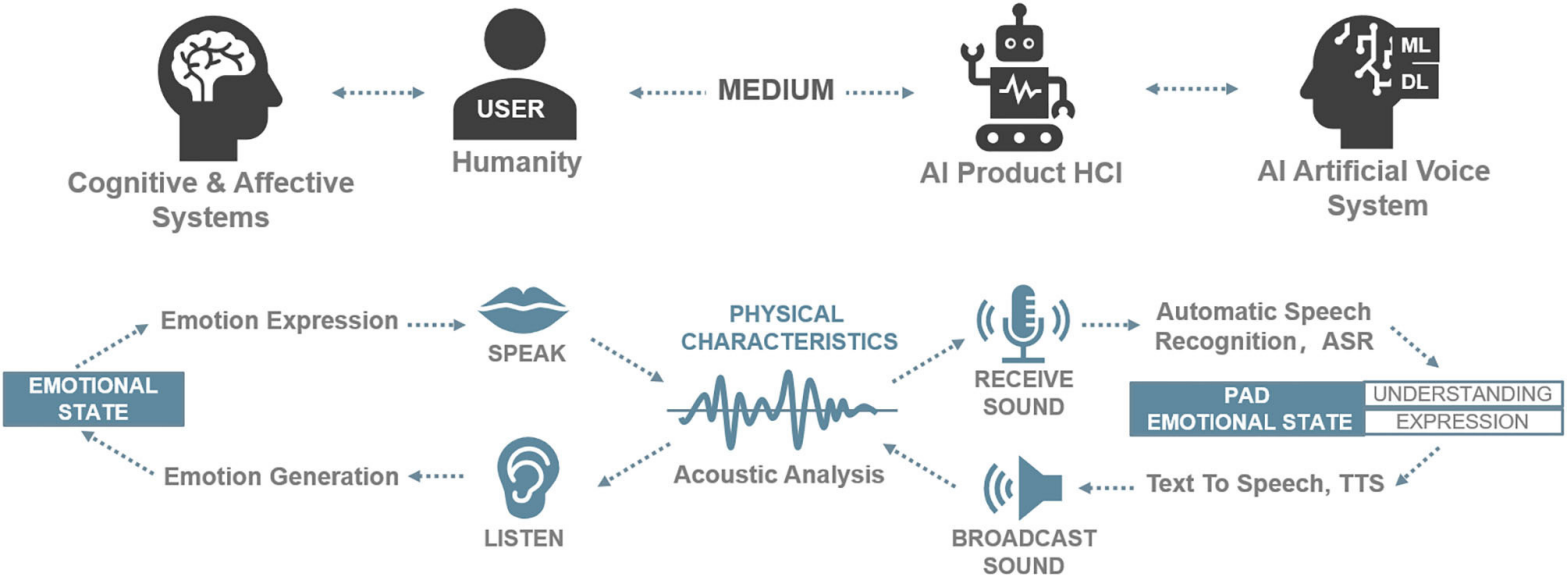
The relationship between artificial acoustic waves and emotional states in the voice interaction systems of intelligent products. Source: Drawn by the authors.

Huttar (1968) further demonstrates that prosodic features of voice play an important role in emotions and suggests simulating these features (e.g., tone, velocity, and volume) in the interface by using artificial voices to express the emotional states of the speaker (Sauter et al., 2010). Subsequently, Professor Picard proposed affective computing (Picard, 2000) and attempted to endow computers with a similar affective mechanism to intelligently understand human emotions in man–machine interactions and, thus, realize effective interactions between an artificial voice and users. It is necessary to gain a subtle understanding of voices using an interdisciplinary approach, including physics and psychology, to understand how to extract and analyze phonetic features (Schwark, 2015; Guo et al., 2020). In addition to the automatic speech recognition (ASR) and text-to-speech (TTS) found in artificial speech, the process involves the emotional analysis of users (Tucker and Jones, 1991; Guo et al., 2020; Hildebrand et al., 2020). In Figure 1, the relationship between artificial acoustic waves and emotional states and the role of artificial acoustic waves in the voice interaction systems of intelligent products are reviewed. Specifically, a user's current emotional state in the PAD model is identified through affective computing according to emotional acoustic features in voice interactions. The user receives responses in an empathic voice expression of the computer in the AI product.

### A Dimensional Framework of the Acoustic Features of Emotions

From a physiological perspective, loosening and contracting the vocal cords leads to rhythm changes in the voice, indicating emotions (Johar, 2016). From the perspective of psychology, relevant studies have proved that prosodic features of voices, such as basic frequency, velocity, and volume, are closely related to any emotional states (Williams and Stevens, 1972; Bachorowski, 1999; Kwon et al., 2003; Audibert et al., 2006; Hammerschmidt and Jürgens, 2007; Sauter et al., 2010; Quinto et al., 2013; Łtowski, 2014; Johar, 2016; Dasgupta, 2017; Hildebrand et al., 2020; Kamiloglu et al., 2020). Murray and Arnott (1993) introduce the concept of utterances and people's emotions, finding three major aspects that influence voice parameters of emotional impacts: utterance timing, utterance pitch contour, and voice quality. Among them, utterance timing and utterance pitch contour are prosodic features. In the past, most studies focused on prosodic features. Although these parameters gave certain differences in emotional distinction, some studies also find disadvantages for intelligent products in judging the emotions of the speaker, including voice quality (spectrum) (Toivanen et al., 2006). Jurafsky and Martin (2014). Experts in both linguistics and computers point out that each acoustic wave can be described completely by the four dimensions of time, frequency, amplitude, and spectrum. Connections between these four dimensions of acoustic waves and emotions in relevant studies are summarized in Table 2.

**Table 2:** Connections between the four dimensions of acoustic features and emotions.

**Dimensions**	**Acoustic features**	**Emotional state correlations**	**Selected research**
Time	Velocity of speech: average time per word (seconds)	anger (+), competence (+), contemplation (–), dominance (–), enthusiasm (+), extraversion (+), fear (+), happiness (+), persuasiveness (+), sadness (–), stress (+), tenderness (–) competence (–), contemplation (+), extraversion (–)	Williams and Stevens, 1972; Miller et al., 1976; Brenner et al., 1994; Tusing and Dillard, 2000; Mohammadi and Vinciarelli, 2012; Dasgupta, 2017
Frequency	Mean Pitch: Fo (Hz)	anger (+), competence (–), confidence (–), empathy (–), extraversion (+), fear (+), happiness (+), nervousness (+), persuasiveness (–), sadness (–), stress (+), tenderness (–), trustworthiness (–)	Williams and Stevens, 1972; Apple et al., 1979; Scherer and Giles, 1979; Brenner et al., 1994; Kwon et al., 2003; Bänziger and Scherer, 2005; Quinto et al., 2013; Bowman and Yamauchi, 2016; Guyer et al., 2019
	Fo *SD*: Pitch variability	anger (+), extraversion (+), happiness (+), sadness (–), shyness (–), sociability (+), tenderness (–)	Apple et al., 1979; Ray, 1986; Burgoon et al., 1990; Abelin and Allwood, 2000; Juslin and Laukka, 2003
Amplitude	Intensity: mean-sones intensity (dB)	aggression (+), anger (+), annoyance (+), dominance (+), extraversion (+), fear (–), happiness (+), tenderness (–), sadness (–), shyness (–), stress (+)	Mallory and Miller, 1958; Scherer and Giles, 1979; Brenner et al., 1994; Johnstone and Scherer, 1999; Kwon et al., 2003; Scherer, 2003; Asutay and Västfjäll, 2012; Quinto et al., 2013
Spectrum	Jitter%: a ratio of variation of fundamental frequency and mean	anger (+), annoyance (+), happiness (+), sadness (–), stress (+)	Johnstone and Scherer, 1999; Li et al., 2007
	Shimmer%: intensity perturbations	anger (+), confidence (+), joy (–), stress (+),	Juslin and Laukka, 2003; Li et al., 2007; Jacob, 2016; Jiang and Pell, 2017
	HNR: Proportion of periodic part and noises in signals (dB)	confidence (+), happiness (+), interest (+), lust (–), pleasure (+)	Jiang and Pell, 2017; Kamiloglu et al., 2020

*Data source: organized in this study*.

The first dimension is *time*, determined by the duration of a vibration from the sound maker (Sueur, 2018; Wayland, 2018) and measured in seconds or milliseconds of acoustic waves. Previous studies explore the influence of gender on velocity. Some studies demonstrate that the velocity of males is higher than females (Feldstein et al., 1993; Verhoeven et al., 2004; Jacewicz et al., 2010); however, most studies on people who speak English find no differences between males and females (Robb et al., 2004; Sturm and Seery, 2007; Nip and Green, 2013). Velocity can indicate the emotional state of the speaker, generally with a high velocity in positive and negative emotional states (e.g., anger, fear, and happiness), but a low velocity in low-wakefulness states (Juslin and Laukka, 2003).

The second dimension is *frequency*, expressed by the number of vibrations of the acoustic wave per second (unit: Hz). The scale of this objective physical quantity corresponds to the fundamental frequency (Fo) of the vocal cord vibrations. Pitch is a subjective psychological quantity of sound, its value determined by the frequency of the acoustic waves (unit: Mel) (Juslin and Laukka, 2003; Colton et al., 2006). Pitch can represent different emotional states. The pitch is increased when a person is feeling anger, happiness, or fear and decreased when a person is sad or bored (Murray and Arnott, 1993; Johar, 2016). With respect to gender, the Fo of a male adult's voice is often lower than a female adult's voice (Mullennix et al., 1995; Pernet and Belin, 2012).

The third dimension is *amplitude*, which determines the intensity of sound (unit: dB). Loudness is the scale of a subjective psychological index of intensity and results from a subjective judgment of a pure tone (unit: phon) (Sueur, 2018; Wayland, 2018). Generally speaking, the loudness of people is about 70 dB (Awan, 1993; Brown et al., 1993). Higher loudness is generally believed to relate to greater dominant traits or aggressiveness (Scherer and Giles, 1979; Abelin and Allwood, 2000; Asutay and Västfjäll, 2012; Yanushevskaya et al., 2013); relatively low loudness indicates people are fearful, sad, or gentle (Johar, 2016). Additionally, males' intensity of sound is slightly higher than that of females (Awan, 1993; Brockmann et al., 2011).

The fourth dimension is *spectrum*, referring to the energy distribution of signals (e.g., voice) in the frequency domain; it is expressed in graphs by analyzing perturbations of acoustic waves or periodic features (Sueur, 2018). The degree of “sound instability” during the formation of voices has been summarized (Hildebrand et al., 2020), reflecting voice quality (Kamiloglu et al., 2020). Vocal jitter is a measure of the periodic variation in fundamental frequency, indicating uneven tones of the speaker. A nervous speaker has instability in the voice (high perturbations) and a quiet speaker has a steady and stable sound (low perturbation) (Farrús et al., 2007; Kamiloglu et al., 2020). Specifically, jitter percentage expresses each basic frequency period's irregularity, that is, the degree of frequency perturbation. It is the ratio between the fluctuations of the fundamental frequency and mean values. A high numerical value indicates that the tone quality is unstable. Shimmer percentage refers to differences in repeated amplitude changes, that is, the degree of amplitude perturbation. It describes the ratio of the mean amplitude variation and respective mean. A high numerical value of shimmer percentage indicates greater changes in sound volume. HNR reflects the ratio of periodic segments and noises in signals (unit: dB). Lower noise energy in voices reflects fewer components of noises and better sound quality (Baken and Orlikoff, 2000; Ferrand, 2007). Some studies have proved that gender has no significant influences on jitter percentage, shimmer percentage, or HNR (Wang and Huang, 2004; Awan, 2006; Brockmann et al., 2008; Ting et al., 2011).

### Research Directions on Connections of Acoustic Features and Emotional States

Studies on the emotional rhythm of voice have pointed out that people's sounds, characterized by pitch, loudness or intensity, and velocity, transfer different emotional information to listeners (Sauter et al., 2010). During a conversation, emotions can be recognized from video clips as short as 60 ms (Pollack et al., 1960; Pell and Kotz, 2011; Schaerlaeken and Grandjean, 2018). The same words and phrases can be expressed differently through fluctuation of different emotional states (Dasgupta, 2017); for example, rumination is related to low velocity and an extended dwell time. Anger is generally related to the loudness of voice (Juslin and Laukka, 2003; Clark, 2005). Fear is related to variations in pitch (Juslin and Laukka, 2003; Clark, 2005). The affective computing team from MIT analyzed variations in acoustic parameters, such as fundamental frequency and duration, during different emotional states; their results show that acoustic features of affective sounds (e.g., happy, surprise, and anger) are similar with the sad acoustic feature being relatively obvious (Sloman, 1999). In brief, the formation of human spoken language involves the interaction of individual traits and emotional states, used as a communication means to understand voices. To recognize and extract information for voice analysis, it is necessary to measure voice quality properties (Johar, 2016; Schaerlaeken and Grandjean, 2018).

To effectively establish an emotional identification and expression system, emotional identification and synthesis based on DL have considerable potential in human–machine interactions (Schuller and Schuller, 2021). Recognizing emotions through the automatic extraction of acoustic features and generating expressions through emotions are the main strategies for relevant research development. It has been proven that a generative adversarial network (GAN) can improve the machine's performance in emotional analysis tasks (Han et al., 2019). Additionally, people begin to think about transfer learning applications in relevant tasks and voice emotional computing modes (Schuller and Schuller, 2021).

Based on the above literature review, research can primarily presently be divided into two types. On the one hand, some studies based on information science strive to gain accurate emotional identification and natural voice expressions through DL. However, these studies lack the explanation for establishing a mathematical model (Ribeiro et al., 2016; Murdoch et al., 2019), thus resulting in the absence of a theoretical foundation for parameter optimization and adjustment. On the other hand, some studies are based on cognitive science and emotional states from the “discrete dimension.” Most of these studies use prosodic features only and have shortages in emotional identification and expression (Toivanen et al., 2006). Studies rarely use the PAD model's emotional states in the intelligent product VUI as the framework for incorporating acoustic features of the spectrum and gender impacts. Hence, interdisciplinary studies are needed to solve the black box problems caused by DL.

## Methods

This study aims to connect humans' emotions and acoustic features from across information, acoustics, and psychology disciplines based on acoustic and cognitive psychology concepts.

### Research Design

Both the purpose of this study and the literature review results have directed the current research to investigate the correlation of two independent variables, namely “gender” and “emotional state.” The emotional state, different from other emotional classification models, considers each emotion has sole coordinates in the PAD space, enabling different emotions to show acoustic features independently. Therefore, the PAD model uses the eight basic emotions for emotional classification and neutral emotions as the benchmark. The dependent variables are seven main features associated with emotional states in the four dimensions of emotional voice sound waves.

### Subjects and Materials

**Figure 2:**
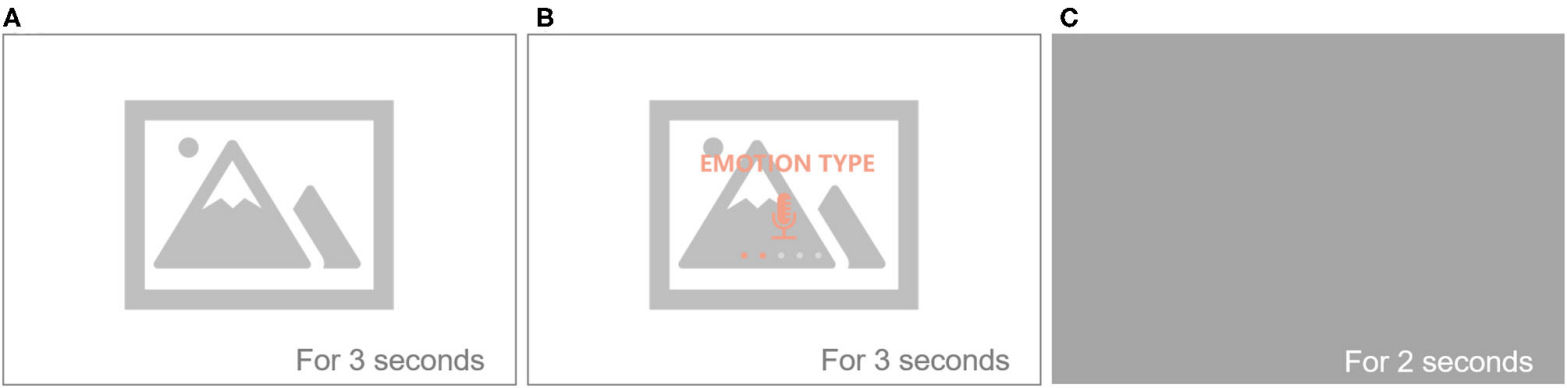
Emotional induction and guidance cases during voice recording of different emotions. **(A)** Emotional stimulus is induced. **(B)** Text to remind the emotion, and then record. Subsequently, **(C)** Interval shady, and then enter the next emotional stimulus to induce. The complete contents are shown in the Appendix: Supplementary Material.

A total of 31 male and 31 female respondents were recruited by the stratified random sampling mode. Respondents have clear cognition with the nine basic emotions of PAD and display explicit oral expression. This study focuses on vocalizations from voice signals, and verbalizations are not transmitted; therefore, the recording of voice data used neutral words and verbalizations transmitted by “
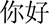
” (Chinese). Because it is easy to induce and simulate emotional recordings that can express real and natural emotions to some extent, PPT was used to provide films as the emotional stimuli to induce and guide recording of the participant (Figure 2). The provided film was confirmed by three relevant experts and then predicted and modified to assure effective induction and prompts.

### Setting and Program of Experiments

**Figure 3:**
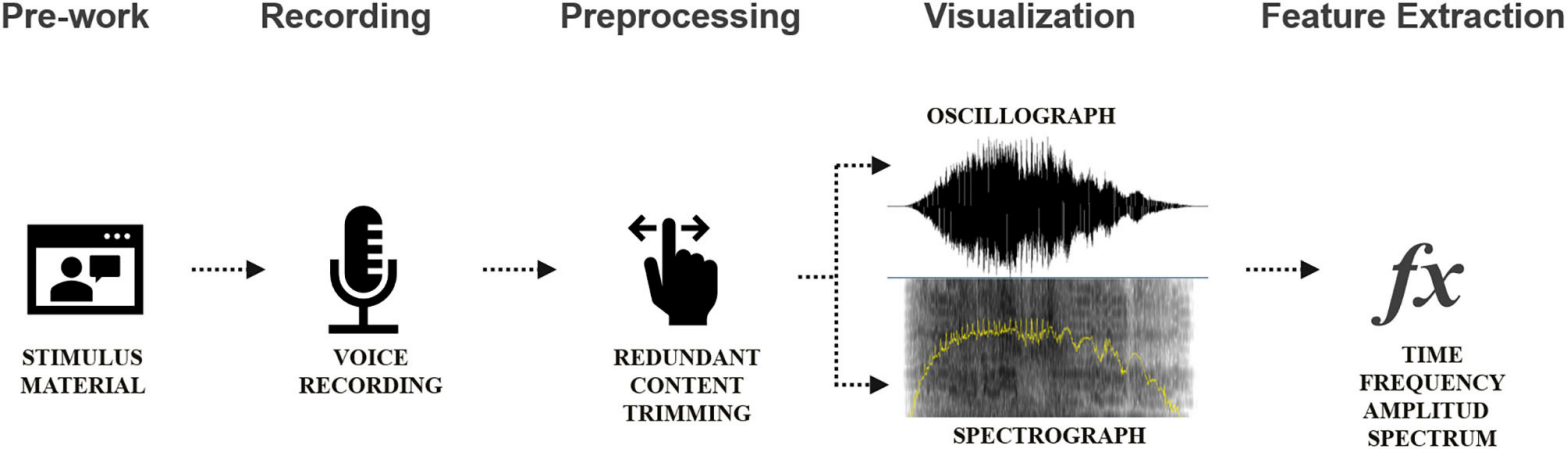
Data collection procedure. Source: drawn by the authors.

**Figure 4:**
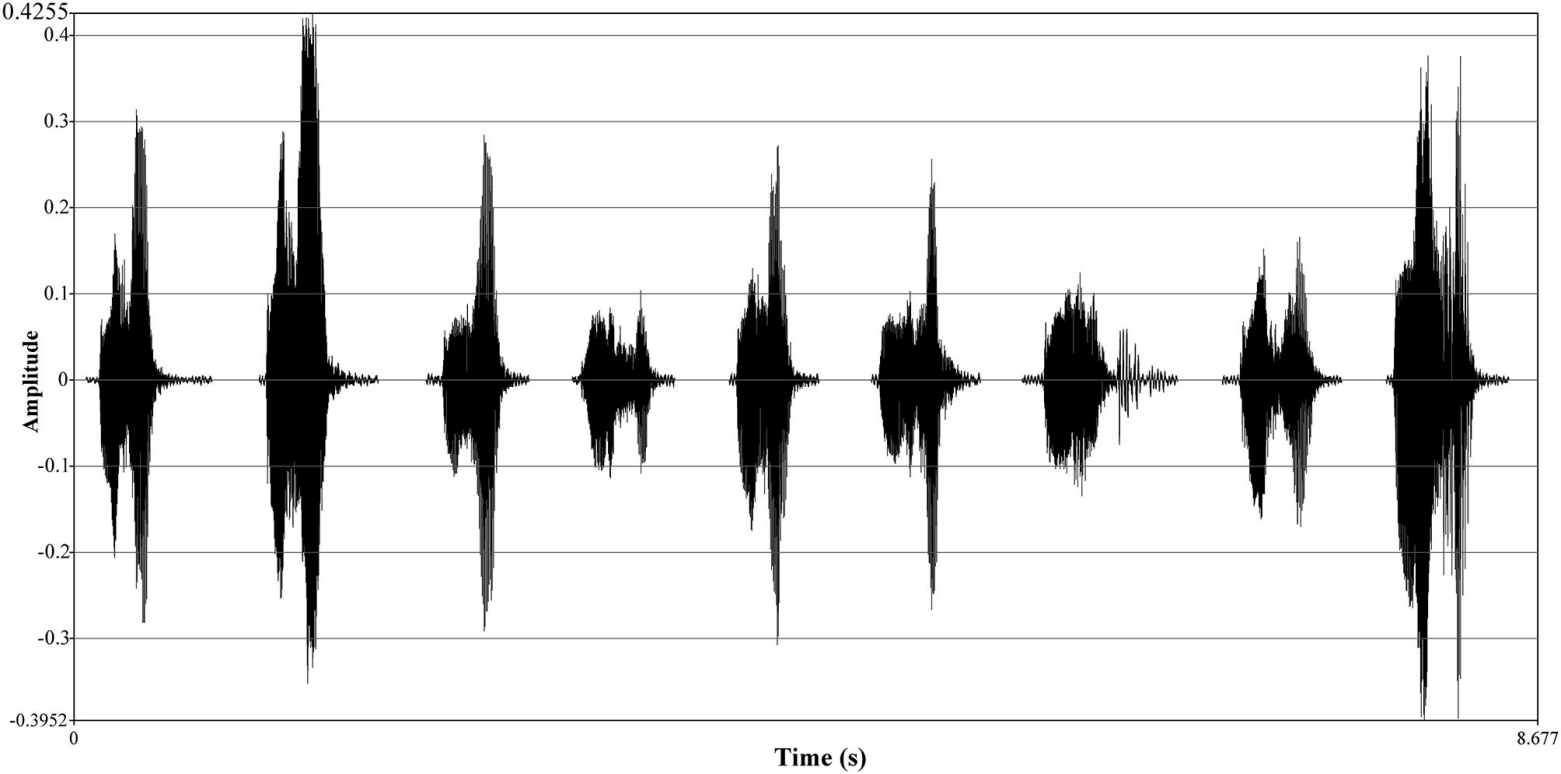
Oscillograph comparison of different emotional voices of respondents. The *Y*-axis of the oscillograph expresses time (unit: s). The *X*-axis, amplitude, has different units of expressions, either decibel (dB) or relative values; it ranges between [−1, 1] and can be expressed by a percentage or frequency value (Sueur, 2018; Wayland, 2018). From the left to the right, a respondent records nine emotions of “
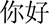
” from ID.1 to ID.9.

Setup of experiments for data acquisition: An empirical study using laboratory experiments was carried out. All respondents engaged in the experiments, and voices were recorded in the same environment using the same settings. The input sound volume was fixed at 70 dB SP. The recording formula was mono channel; sampling frequency: 44.1 kHz; and resolution: 16 bits and WAV file. The relevant program is shown in Figures 3, 4.

**Figure 5:**
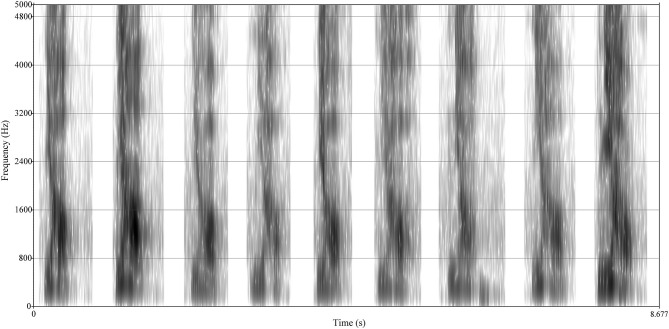
Comparison of spectrographs of respondents among different emotions. The *Y*-axis of the spectrograph is the same as the waveform and expresses the amplitude. The *X*-axis represents frequency (unit: Hz). The frequency spectrum is the variation of voice energy with frequency. In addition, different amplitudes (or loudness) were expressed by the color gradient of data points.

The audio recording process: First, selected respondents, in the closed experimental space without disturbance, were introduced to the experimental process and audition by the same prompts. Second, respondents wore a headset microphone in a closed space, and a provided laptop played the stimulus and prompted the film using Adobe Audition 2019. Respondents provided data of nine emotions: neutral, exuberant, bored, dependent, disdainful, relaxed, anxious, docile, and hostile. The content of the audio recordings from each respondent was then confirmed, and residual contents were preprocessed, including polishing and numbering. Finally, acoustic features were analyzed using the Praat 6.13 voice software (Figure 5).

Analysis of the spectrum was done using the calculation formulas of jitter percentage, shimmer percentage, and HNR as outlined below (Boersma, 1993; Fernandes et al., 2018; Sueur, 2018). Nine emotional voices were selected and analyzed by Praat, and characteristic parameter data of seven emotional voices were directly extracted.

In phonetics, jitter reflects the fast repeated changes of the fundamental frequency, and it primarily describes the variation amplitude of any fundamental frequency. As shown below,

(1) $$\begin{array}{r} jitter_{absolute}=\overset{N}{\underset{i=2}{\sum }}\;|T_{i}-T_{i-1}\;/\;\left(N-1\right) \end{array}$$

*T*_i_ is the duration of the pitch period *i* (unit: ms), and *N* is the quantity of all pitch periods. Jitter_absolute_ calculates the absolute mean of differences between any two adjacent pitch periods. The mean period is calculated using

(2) $$\begin{array}{r} meanPeriod=\overset{N}{\underset{i=1}{\sum }}T_{i}/N \end{array}$$

The jitter percentage is calculated using

(3) $$\begin{array}{r} jitter\%=jitter_{absolute}/meanPeriod \end{array}$$

The *jitter*_*absolute*_ is divided by the *meanPeriod*, deriving the ratio between perturbation of fundamental frequency and mean during the pronunciation.

### Calculation of Shimmer Percentage

Shimmer percentage reflects changes of amplitude among different periods and is calculated using

(4) $$\begin{array}{r} shimmer_{absolute}=\;\overset{N}{\underset{i=2}{\sum }}|A_{k}-\;A_{k-1}|/\;\left(N-1\right)\; \end{array}$$

(5) $$\begin{array}{r} meanShimmer=\;\overset{N}{\underset{i=1}{\sum }}A_{k}/N \end{array}$$

(6) $$\begin{array}{r} Shimmer\%=\;shimmer_{absolute}/meanShimmer \end{array}$$

The mean of amplitude changes between two adjacent periods is calculated from *shimmer*_*absolute*_. The *Shimmer%* is the ratio between the mean variation of amplitudes and the average value.

### Calculation of HNR

HNR refers to the ratio of the periodic and noise parts in speech signals, and it primarily reflects the hoarse degree of voices. The calculation used to determine HNR is explained below.

The autocorrelation function (*r*(*x*)) of the voice delay signal x is defined as

(7) r(x)=∫s(t)2(t+X)dt

where *s*(*t*) is the stable time signal, and the function achieves the global maximum when *x* = 0. If the function has global maximum points at other moments in addition to *x* = 0, a period of *T*_0_ *is assumed*. For any positive integer (*n*), then

(8) $$\begin{array}{r} r\left(nT_{0}\right)=\;r\left(0\right) \end{array}$$

If no other global maximum points in addition *to x* = 0 are detected, then other local maximum points may exist, where

(9) $$\begin{array}{r} r^{\prime }\left(\tau \right)=\;r_{x}\left(x\right)/r\left(0\right) \end{array}$$

*s*(*t*) is defined as the periodic signal with a period of *T*_0_, and N(t) is a noise signal. At *x* = 0, the voice signal is *r*(0) = *T*_*H*_(0) + *T*_*N*_(0). As *r*(0) = *r*_*H*_(0) + *r*_*N*_(0), the following equations can be applied:

(10) $$\begin{array}{r} r^{\prime }\left(X_{\max}\right)=r_{H}\left(0\right)/r\left(0\right) \end{array}$$

(11) $$\begin{array}{r} 1-r^{\prime }\left(X_{\max}\right)=r_{H}\left(0\right)/r\left(0\right) \end{array}$$

$r^{\prime }\left(X_{\max}\right)$ describes the size of the relative energy of periodic parts in the voice signals and its complementary set $1-r^{\prime }\left(X_{\max}\right)$ describes the size of the relative energy of noises in the voice signal. HNR can be further defined as

(12) $$\text{HNR }(\text{in dB})=10\;*\;log_{10}\frac{r_{\;x}^{\prime }(\tau_{\max})}{1-r_{\;x}^{\prime }(\tau_{\max})}$$

The function has a global maximum when τ = 0, where x(t) is a steady time signal and a global maximum when τ = 0.

## Results

The extracted seven-feature data of different emotions of different genders were analyzed using SPSS V.26 to conduct a two-way ANOVA, mixed design. Gender was used as the independent variable, and emotional state was used as the dependent variable to understand the variation in seven acoustic features of different genders under different emotions.

### General Conditions of Respondents

A total of 62 respondents, including 31 males and 31 females, were recruited. These participants can be grouped according to age: 21–30 years old: nine females and eight males; 31–40 years old: eight females and eight males; 41–50 years old: eight females and eight males; and 51–60 years old: six females and seven males.

### Difference Test Analysis of the Acoustic Parameters

To show significant differences in acoustic features under different emotions and gender, the same respondents were repeatedly measured, testing the seven acoustic features of emotions. Results of the correlation analyses are shown below.

**Velocity:** relevant data of seconds per word are listed in Tables 3, 4.

**Table 3:** Fine grids and marginal means of emotional states and gender on acoustic features.

		**Gender**		**Marginal means**
		**Female**	**Male**	
State	Neutral	0.29	0.26	0.28
	Exuberant	0.28	0.24	0.26
	Bored	0.52	0.48	0.50
	Dependent	0.38	0.36	0.37
	Disdainful	0.29	0.25	0.27
	Relaxed	0.34	0.30	0.32
	Anxious	0.26	0.21	0.23
	Docile	0.33	0.28	0.31
	Hostile	0.28	0.24	0.26
Marginal means		0.33	0.29	0.29

**Table 4:** Two-way ANOVA abstract of emotional states and gender on velocity.

**Variable**	***SS***	***Df***	***MS***	***F***	***post hoc* comparisons**
Gender	0.24	1	0.24	2587.77^***^	Female > Male
State_b_	3.37	2.53	1.33	76.37^***^	(1) > (2); (2) > (7); (3) > (1)-(9); (4) > (1)-(2), (6)-(9); (5) > (7), (9); (6) > (1)-(2), (5), (7), (9); (8) > (1)-(2), (5), (7), (9); (9) > (7);
Gender X state	0.01	2.53	0.00	0.25	
Block	1.24	60	0.02		
Error	2.65	151.75	0.02		

*It indicates that b is the interval design factor (dependent factor).*

*(1) neutral (2) exuberant (3) bored (4) dependent (5) disdainful (6) relaxed (7) anxious (8) docile (9) hostile*.

*** *p < 0.001*.

The interaction tests for gender and emotional state (*SS* = 0.01; *Df* = 2.53; *MS* = 0.00; *F* = 0.25; *P* > 0.05) did not yield any significant results, i.e., participants' velocity in expressing the nine different emotions was not significantly correlated to gender.

**Gender main effect:** The influence of velocity on overall emotional states varies significantly between males and females (*F* = 2587.76, *p* < 0.05). The velocity (*M* = 0.33) of female respondents under different emotional states is significantly lower than that of males (*M* = 0.29).

**State main effect:** Velocity under different emotional states varies significantly for the overall factor, gender (*F* = 76.37, *p* < 0.05). According to the multiple comparison, the state anxious (*M* = 0.23) shows the highest velocity, followed by exuberant and hostile (*M* = 0.26), disdainful (*M* = 0.27), neutral (*M* = 0.28), docile (*M* = 0.31), relaxed (*M* = 0.32), dependent (*M* = 0.37), and bored (*M* = 0.5), successively.

**Fo (Hz):** The interaction test showed significant results for both gender and emotional state (*SS* = 72887.47; *Df* = 1.85; *MS* = 39437.31; *F* = 15.90; *p* < 0.05; ω^2^ = 0.21), i.e., participants' Fo (Hz) varied across gender and emotional state. Relevant data abstracts of mean pitch are listed in Table 5.

**Table 5:** Test of simple main effect in the mixed design of gender and emotional state in Fo.

**Variable**	***SS***	***Df***	***MS***	***F***	***post hoc* comparisons**
**State**					
Female	621352.46	1.59	391784	111.30^***^	(1) > (4); (2) > (1)-(6), (9); (3) > (5); (4) > (5); (6) > (1), (3)-(5); (7) > (1), (3)-(6), (9); (8)> (1), (3)-(6), (9); (9) > (1), (3)-(5).
Male	372745.37	1.54	241754	103.96^***^	(1) > (4); (2) > (1)-(6), (8), (9); (3) > (4); (5) > (1), (3), (4); (6) > (1), (3)-(5), (8)-(9); (7) > (1), (3)-(9); (8) > (1). (3)-(5), (9); (9) > (1). (3)-(5).
**Gender**					
Neutral	93367.55	1	93367.55	198.83^***^	Female (*M* = 214.73) > Male (*M* = 137.12)
Exuberant	143410.15	1	143410.15	113.02^***^	Female (*M* = 314.45) > Male (*M* = 218.26)
Bored	100950.05	1	100950.05	147.32^***^	Female (*M* = 214.73) > Male (*M* = 134.03)
Dependent	142056.14	1	142056.14	324.47^***^	Female (*M* = 215.45) > Male (*M* = 118.72)
Disdainful	39185.22	1	39185.22	49.67^***^	Female (*M* = 188.35) > Male (*M* = 138.03)
Relaxed	89769.08	1	89769.08	51.28^***^	Female (*M* = 283.05) > Male (*M* = 205.95)
Anxious	129113.74	1	129113.74	43.98^***^	Female (*M* = 309.57) > Male (*M* =218.30)
Docile	290924.98	1	290924.98	66.71^***^	Female (*M* = 309.00) > Male (*M* = 171.10)
Hostile	188635.07	1	188635.07	207.12^***^	Female (*M* = 279.94) > Male (*M* = 169.62)

*(1) neutral (2) exuberant (3) bored (4) dependent (5) disdainful (6) relaxed (7) anxious (8) docile (9) hostile*.

*** *p < 0.001*.

**Gender simple main effect:** Females show significantly different effects of Fo on emotional states (*F* = 111.30, *p* < 0.05), according to the results of *post hoc* comparisons: (1) > (4); (2) > (1)–(6), (9); (3) > (5); (4) > (5); (6) > (1), (3) (5); (7) > (1), (3)–(6), (9); (8)> (1), (3)–(6), (9); (9) > (1), (3)–(5). Males (*F* = 103.96, *p* < 0.05) also show differences, according to results of *post hoc* comparisons: (1) > (4); (2) > (1)–(6), (8), (9); (3) > (4); (5) > (1), (3), (4); (6) > (1), (3)–(5), (8)–(9); (7) > (1), (3)–(9); (8) > (1). (3)–(5), (9); (9) > (1). (3)–(5). These results demonstrate that ranks of emotional states are different between males and females.

**State simple main effect:** With respect to influences of Fo (Hz) on gender under different emotional states, F-values of neutral, exuberant, bored, dependent, relaxed, disdainful, anxious, docile, and hostile states are 198.83, 113.02, 147.32, 324.47, 49.67, 51.28, 43.98, 66.71, and 207.12, respectively (*p* < 0.05). According to the results of *post hoc* comparisons, females have a significantly higher Fo than males.

**Fo SD:** The interaction test was significant across gender and emotional state (*SS* = 13144.75; *Df* = 3.67; *MS* = 3586.29; *F* = 10.80; *p* < 0.05; ω^2^ = 0.15), i.e., participants' Fo *SD* varied across gender and emotional state. Relevant data abstracts of pitch variability are listed in Table 6.

**Table 6:** Simple main effect test of mixed design of gender and emotional states in Fo *SD*.

**Variable**	***SS***	***Df***	***MS***	***F***	***post hoc* comparisons**
**State**					
Female	19634.95	2.75	7153.90	24.69^***^	(1) > (4)-(6); (2) > (1), (4)-(8); (3) > (1), (4)-(8); (6) > (4); (7) > (1), (5); (8) > (4)-(6); (9) > (1), (4)-(8).
Male	3935.73	2.45	1605.58	2.43	
**Gender**					
Neutral	53.16	1	53.16	0.17	
Exuberant	2462.42	1	2462.42	47.88^***^	Female (*M* = 33.86) > male (*M* = 21.25)
Bored	8920.32	1	8920.32	92.90^***^	Female (*M* = 37.20) > male (*M* =13.21)
Dependent	858.95	1	858.95	9.52^**^	Male (*M* =20.75) > Female (*M* = 13.30)
Disdainful	2.74	1	2.74	0.01	
Relaxed	381.33	1	381.33	3.92	
Anxious	17.25	1	17.25	0.08	
Docile	11.01	1	11.01	0.15	
Hostile	6328.66	1	6328.66	15.38^***^	Female (*M* = 37.57) > male (*M* = 17.36)

*(1) neutral (2) exuberant (3) bored (4) dependent (5) disdainful (6) relaxed (7) anxious (8) docile (9) hostile*.

** *p < 0.01;*

*** *p < 0.001*.

**Gender simple main effect:** Females show significantly different effects of Fo *SD* on emotional states (*F* = 2.43, *p* > 0.05), according to the results of *post hoc* comparisons: (1) > (4)–(6); (2) > (1), (4)–(8); (3) > (1), (4)–(8); (6) > (4); (7) > (1), (5); (8) > (4)–(6); (9) > (1), (4)–(8). Males (*F* = 2.43, *p* > 0.05) show no significant differences.

**State simple main effect:** Concerning influences of Fo *SD* on gender under different emotional states, *F* values of exuberant, bored, dependent, and hostile states are 47.88, 92.90, and 9.52, respectively (*p* < 0.05). According to the results of *post hoc* comparisons, females give significantly higher values than males; however, males > females with respect to the dependent variable.

**Intensity (dB):** The interaction test was significant across gender and emotional state (*SS* = 7624.57; *Df* = 1.99; *MS* = 314.08; *F* = 9.25; *p* < 0.05; ω^2^ = 0.13), i.e., participants' intensity varied across gender and emotional state. Relevant data abstracts of mean-sones intensity are listed in Table 7.

**Table 7:** Simple main effect test using mixed design of gender and emotional states on intensity (dB).

**Variable**	***SS***	***Df***	***MS***	***F***	***post hoc* comparisons**
**State**					
Female	5071.70	1.41	3596.94	64.11^***^	(1) > (3)-(4), (7)-(8); (2) > (1)-(8); (4) > (3); (5) > (1), (3)-(4), (7)-(8); (6) > (1), (3)-(4), (7)-(8); (7) > (3); (8) > (3); (9) > (1), (2) -(8).
Male	2939.66	2.26	1300.21	52.60^***^	(1) > (3)-(4), (7); (2) > (1), (3)-(9); (3) > (7); (4) > (7); (5) > (1), (3), (4), (7); (6) > (1), (4)-(8); (8) > (3), (4), (7); (9) > (3), (4), (5), (7), (8).
**Gender**					
Neutral	151.32	1	151.32	3.55	
Exuberant	143.85	1	143.85	2.20	
Bored	820.46	1	820.46	17.46^***^	Male (*M* = 69.65) > Female (*M* = 62.38)
Dependent	363.48	1	363.48	8.23^**^	Male (*M* = 69.52) > Female (*M* = 64.68)
Disdainful	80.62	1	80.62	1.24	
Relaxed	261.91	1	261.91	3.58	
Anxious	45.29	1	45.29	1.42	
Docile	546.12	1	546.12	9.88^**^	Male (*M* = 71.71) > Female (*M* = 65.78)
Hostile	0.15	1	0.15	0.00	

*(1) neutral (2) exuberant (3) bored (4) dependent (5) disdainful (6) relaxed (7) anxious (8) docile (9) hostile*.

** *p < 0.01;*

*** *p < 0.001*.

**Gender simple main effect:** Both males and females show significantly different effects of intensity (dB) on emotional states: Females (*F* = 64.11, *p* < 0.05) and males (*F* = 52.60, *p* < 0.05). According to *post hoc* comparisons, results of females are (1) > (3)–(4), (7)–(8); (2) > (1)–(8); (4) > (3); (5) > (1), (3)–(4), (7)–(8); (6) > (1), (3)–(4), (7)–(8); (7) > (3); (8) > (3); (9) > (1), (2)–(8). Results of males are (1) > (3)–(4), (7); (2) > (1), (3)–(9); (3) > (7); (4) > (7); (5) > (1), (3), (4), (7); (6) > (1), (4)-(8); (8) > (3), (4), (7); (9) > (3), (4), (5), (7), (8). The results demonstrate that ranks of emotional states are different between males and females.

**State simple main effect:** Concerning influences of intensity (dB) on gender under different emotional states, F-values of bored, dependent, and docile are 17.46, 8.23 and 9.88, respectively (*p* < 0.05). According to the results of *post hoc* comparisons, males give significantly higher results than females.

**Jitter%:** The interaction test resulted in significant outcomes considering gender and emotional state (*S* = 230.33; *Df* = 2.60; *MS* = 88.67; *F* =32.05; *p* < 0.05; ω^2^ = 0.35), i.e., participants' Jitter% varied across gender and emotional state. Relevant data abstracts of the ratio between the fundamental frequency changes and the mean are listed in Table 8.

**Table 8:** Simple main effect test of mixed design of gender and emotional states in Jitter%.

**Variable**	***SS***	***Df***	***MS***	***F***	***post hoc* comparisons**
**State**					
Female	78.25	2.66	29.372	25.87^***^	(1) > (2), (8); (3) > (2), (8); (4) > (2), (8); (5) > (2), (8)-(9); (6) > (1)-(5), (7)-(9); (7) > (1)-(5), (8)-(9).
Male	419.94	1.88	223.091	37.01^***^	(1) > (2)-(6), (9); (2) > (5)-(6), (9); (4) > (3)-(6), (9); (7) > (1)-(6), (8)-(9); (8) > (2)-(6), (9).
**Gender**					
Neutral	78.81	1	78.81	82.90^***^	Male (*M* = 4.19) > Female (*M* = 1.93)
Exuberant	16.00	1	16.00	63.04^***^	Male (*M* = 2.59) > Female (*M* = 1.57)
Bored	2.01	1	2.01	8.11^***^	Male (M = 2.33) > Female (*M* = 1.97)
Dependent	11.24	1	11.24	14.52^***^	Male (*M* = 2.89) > Female (*M* = 2.04)
Disdainful	0.01	1	0.01	0.02	
Relaxed	18.76	1	18.76	23.77^***^	Female (*M* = 3.18) > Male (*M* = 2.08)
Anxious	124.25	1	124.25	35.51^***^	Male (*M* = 5.70) > Female (*M* = 2.87)
Docile	130.76	1	130.76	65.22^***^	Male (*M* = 2.10) > Female (*M* = 1.75)
Hostile	1.86	1	1.86	2.61	

*Note. (1) neutral (2) exuberant (3) bored (4) dependent (5) disdainful (6) relaxed (7) anxious (8) docile (9) hostile*.

*** *p < 0.001*.

**Gender simple main effect:** With respect to Jitter% of males and females under different emotional states, females (*F* = 25.87, *p* < 0.05) and males (*F* = 37.01, *p* < 0.05) both have significant effects. According to *post hoc* comparisons, females show (1) > (2), (8); (3) > (2), (8); (4) > (2), (8); (5) > (2), (8)-(9); (6) > (1)–(5), (7)–(9); (7) > (1)–(5), (8)–(9). Males show (1) > (2)–(6), (9); (2) > (5)–(6), (9); (4) > (3)–(6), (9); (7) > (1)–(6), (8)–(9); (8) > (2)–(6), (9). These results demonstrate that ranks of emotional states are different between males and females.

**State simple main effect:** Concerning influences of Jitter% on gender under different emotional states, F-values of neutral, exuberant, bored, dependent, relaxed, anxious, and docile are 82.90, 63.04, 8.11, 14.52, 23.77, 35.51, and 65.22, respectively (*p* < 0.05). According to the results of *post hoc* comparisons, females > males for relaxed and males > females for the remaining six emotional states.

**Shimmer%:** The interaction test yielded significant results considering gender and emotional state (*S* = 1712.65; *Df* = 4.29; *MS* = 399.46; *F* = 49.4; *p* < 0.05; ω^2^ = 0.45), i.e., participants' Shimmer % varied across gender and emotional state. Relevant data abstracts of intensity perturbations are listed in Table 9.

**Table 9:** Simple main effect test of mixed design of gender and emotional states in Shimmer%.

**Variable**	***SS***	***Df***	***MS***	***F***	***post hoc* comparisons**
**State**					
Female	7026.62	2.69	2617.24	240.70^***^	(1) > (2), (4), (8)-(9); (2) > (8)-(9); (3) > (2), (4), (8)-(9); (4) > (8)-(9); (5) > (1)-(4), (8)-(9); (6) > (1)-(4), (8)-(9); (7) > (1)-(4), (8)-(9); (8) > (9).
Male	9662.34	3.42	2825.46	241.26^***^	(1) > (2)-(9); (2) > (8)-(9); (3) > (2), (8)-(9); (4) > (2)-(3), (6), (8)-(9); (5) > (2), (6), (8)-(9); (6) > (8)-(9); (7) > (2)-(9); (8) > (9).
**Gender**					
Neutral	1276.10	1	1276.10	184.14^***^	Male (*M* = 18.73) > Female (*M* = 9.65)
Exuberant	22.72	1	22.72	5.78^*^	Male (*M* = 9.24) > Female (*M* = 8.03)
Bored	320.89	1	320.89	7.45^**^	Male (*M* =11.12) > Female (*M* = 9.84)
Dependent	320.89	1	320.89	45.81^***^	Male (*M* = 12.93) > Female (*M* = 8.38)
Disdainful	218.49	1	218.49	19.99^***^	Female (*M* = 15.55) > Male (*M* = 11.79)
Relaxed	50.92	1	50.92	6.48^*^	Female (*M* = 12.30) > Male (*M* =10.48)
Anxious	94.24	1	94.24	9.02^**^	Male (*M* = 15.81) > Female (*M* = 13.34)
Docile	0.05	1	0.05	11.33^**^	Male (*M* = 0.33) > Female (*M* = 0.28)
Hostile	0.03	1	0.03	7.58^**^	Male (*M* =.28) > Female (*M* = 0.24)

*(1) neutral (2) exuberant (3) bored (4) dependent (5) disdainful (6) relaxed (7) anxious (8) docile (9) hostile*.

* *p < 0.05;*

** *p < 0.01;*

*** *p < 0.001*.

**Gender simple main effect:** With respect to Shimmer% of males and females under different emotional states, females (*F* = 240.70, *p* < 0.05) and males (*F* = 241.26, *p* < 0.05) both have significant effects. According to *post hoc* comparisons, females show (1) > (2), (4), (8)–(9); (2) > (8)-(9); (3) > (2), (4), (8)-(9); (4) > (8)–(9); (5) > (1)–(4), (8)–(9); (6) > (1)–(4), (8)–(9); (7) > (1)–(4), (8)–(9); (8) > (9). Males show (1) > (2)–(9); (2) > (8)–(9); (3) > (2), (8)–(9); (4) > (2)–(3), (6), (8)–(9); (5) > (2), (6), (8)–(9); (6) > (8)–(9); (7) > (2)–(9); (8) > (9). These results demonstrate that ranks of emotional states are different between males and females.

**State simple main effect:** Concerning influences of Shimmer% on gender under different emotional states, *F*-values of neutral, exuberant, bored, dependent, disdainful, relaxed, anxious, docile, and hostile are 82.90, 63.04, 8.11, 14.52, 19.99, 23.77, 35.51, 65.22, and 7.58, respectively (*p* < 0.05). According to *post hoc* comparison results, females are significantly higher than males concerning disdainful and relaxed, which is the opposite of the remaining emotional states.

**HNR:** The interaction test yielded significant results considering gender and emotional state (*SS* = 1071.63; *Df* = 3.76; *MS* = 284.69; *F* = 37.42; *p* < 0.05; ω^2^ = 0.38), i.e., participants' HNR varied across gender and emotional state. Relative data abstracts of the ratio of periodic part and noise in signals are listed in Table 10.

**Table 10:** Simple main effect test of mixed design of gender and emotional states in HNR.

**Variable**	***SS***	***Df***	***MS***	***F***	***post hoc* comparisons**
**State**					
Female	1500.08	1.99	754.38	45.87^***^	(1) > (3), (5)-(7); (2) > (1), (3)-(7); (3) > (6)-(7); (4) > (3), (5)-(7); (5) > (6)-(7); (8) > (1)-(7); (9) > (1), (3)-(7).
Male	759.60	2.92	259.89	30.90^***^	(2) > (1), (7); (3) > (1)-(2), (4)-(5), (7)-(9); (4) > (1); (5) > (1), (7); (6) > (1)-(2), (4), (7)-(9); (8) > (1)-(2), (7); (9) > (1)-(2), (7).
**Gender**					
Neutral	369.81	1	369.81	62.35^***^	Female (*M* =13.08) > Male (*M* = 8.19)
Exuberant	261.34	1	261.34	40.59^***^	Female (*M* =15.14) > Male (*M* = 11.03)
Bored	38.21	1	38.21	8.50^**^	Male (*M* = 12.99) > Female (*M* = 11.42)
Dependent	65.90	1	65.90	18.12^***^	Female (*M* =13.54) > Male (*M* = 11.48)
Disdainful	3.56	1	3.56	0.44	
Relaxed	246.12	1	246.12	49.74^***^	Male (*M* = 13.09) > Female (*M* = 9.10)
Anxious	10.16	1	10.16	4.02	
Docile	210.75	1	210.75	22.60^***^	Female (*M* =15.97) > Male (*M* = 12.29)
Hostile	132.10	1	132.10	15.43^***^	Female (*M* =14.93) > Male (*M* = 12.01)

*(1) neutral (2) exuberant (3) bored (4) dependent (5) disdainful (6) relaxed (7) anxious (8) docile (9) hostile*.

** *p < 0.01;*

*** *p < 0.001*.

**Gender simple main effect:** With respect to HNR of males and females under different emotional states, females (*F* = 45.87, *p* < 0.05) and males (*F* = 30.90, *p* < 0.05) both show a significant effect. According to *post hoc* comparisons, females show (1) > (3), (5)–(7); (2) > (1), (3)–(7); (3) > (6)–(7); (4) > (3), (5)–(7); (5) > (6)–(7); (8) > (1)–(7); (9) > (1), (3)–(7). Males show (2) > (1), (7); (3) > (1)–(2), (4)–(5), (7)–(9); (4) > (1); (5) > (1), (7); (6) > (1)–(2), (4), (7)–(9); (8) > (1)–(2), (7); (9) > (1)–(2), (7). These results demonstrate that ranks of emotional states are different between males and females.

**State simple main effect:** With respect to influences of HNR on gender under different emotional states, F-values of neutral, exuberant, bored, dependent, relaxed, docile, and hostile are 62.35, 40.59, 8.50, 18.12, 49.74, 22.60, and 15.43, respectively (*p* < 0.05). According to the results of *post hoc* comparisons, males give significantly higher values than females in terms of bored and relaxed although the opposite phenomenon is observed for the remaining five emotional states.

## Discussion And Conclusions

This study focuses on physical quantities of acoustic features and their differences according to gender and the emotional states of the PAD model during emotion–voice interactions of AI. The study found significant differences in users' gender and emotional states of the PAD model with respect to seven major acoustic features: (1) With respect to gender and emotional states, Fo (Hz), Fo *SD*, intensity (dB), Jitter%, Shimmer%, and HNR have interactions, and velocity displays no interaction. (2) There are significant gender differences in terms of velocity of eight emotional states in PAD. Moreover, males show significantly higher velocity (*M* = 0.29) compared to females (*M* = 0.33). (3) Males show no significant differences in six of the acoustic features, except Fo *SD*. Looking at the gender simple main effect, there are significant gender differences in terms of degree and ranking of emotional states. Looking at the state simple main effect, Fo (Hz) shows significant differences among different emotional states. Fo *SD* is significantly different in terms of exuberant, bored, dependent, and hostile states. Intensity (dB) is significantly different with respect to bored, dependent, and docile states. There are significant differences in Jitter% in neutral, exuberant, bored, dependent, relaxed, anxious, and docile states. Shimmer% has significant differences. HNR presents significant differences in neutral, exuberant, bored, dependent, relaxed, docile, and hostile states. The above analyses found physical quantities of relevant parameters and rankings as shown in the results. Specifically, the voice-affective interaction of intelligent products was used as the preset scene. Therefore, the PAD model is different in terms of emotional classification from the emotional classification found in the literature review (Williams and Stevens, 1972; Johnstone and Scherer, 1999; Abelin and Allwood, 2000; Quinto et al., 2013; Bowman and Yamauchi, 2016; Dasgupta, 2017; Hildebrand et al., 2020). Moreover, some acoustic features are different, and it is impossible to compare directly. Directionality of classification is compared with research results, which has not been investigated in past empirical studies; however, there are significant differences in rhythms of different emotions. For gender, previous studies mainly found that men speak more quickly than women (Feldstein et al., 1993; Verhoeven et al., 2004; Jacewicz et al., 2010), but it has also been found that there is no significant difference between men and women (Robb et al., 2004; Sturm and Seery, 2007; Nip and Green, 2013). This study further compared expressions of emotional states and concluded that men speak more quickly than women.

We comprehensively explored the influence of eight emotional states of the PAD model and gender on affective recognition and expression of acoustic features (e.g., velocity, Fo, frequency spectra) in a systematic method. In terms of theoretical implications, the PAD model of intelligent products provides an emotional model that is different from previously used models. In emotional computing, the PAD model is conducive to understanding the influences of gender and emotional states on the connection between acoustic features and psychology in AI affective-voice interaction, including physical variables and their differences. This aids in understanding the acoustic features of affective recognition and expression. In terms of practical applications, in view of the development trends of intelligent products on the market, man–machine interaction will be popularized in intelligent-home life, travel, leisure, entertainment, education, and medicine in the future. This study will help to improve the affective-voice interaction scenes of intelligent products and connections between the emotional states and acoustic features of the speaker. The analysis of acoustic features under different emotions and genders provides an empirical foundation for adjusting the parameters of the affective-voice interaction mathematical models and offsets limitations of current deep learning acoustic models' “explanatory” power. The research results can provide a reference for the adjustment of model parameters during optimization of affective recognition and affective expression.

This study was designed for theoretical and practical application; however, the recorded voices only used Chinese materials. There may be some differences with different languages, which deserves particular attention for generalization of the results. Subsequent studies can further investigate correlations between emotional classification of PAD and voice rhythm of different genders in the PAD model to provide a theoretical basis and supplement shortages of deep learning. This study aims to strengthen emotional integration during man–machine interaction, allowing users and products to generate the empathy effect and, thus, expand the human–computer relationship and highlighting the value of products.

## Data Availability Statement

The original contributions presented in the study are included in the article/Supplementary Material, further inquiries can be directed to the corresponding author/s.

## Ethics Statement

Ethical review and approval was not required for the study on human participants in accordance with the local legislation and institutional requirements. Written informed consent for participation was not required for this study in accordance with the national legislation and the institutional requirements.

## Author Contributions

K-LH: conceptualization and writing. K-LH and S-FD: methodology and formal analysis. K-LH and XL: investigation. S-FD and XL: resources. K-LH: organized the database and analyzed and interpreted the data.

## Conflict of Interest

The authors declare that the research was conducted in the absence of any commercial or financial relationships that could be construed as a potential conflict of interest.
